# Supplementary material for: Antibiotic Use during Pregnancy in South Korea Using 2011–2020 National Health Insurance Claims Data
Source: Antibiotics (Basel). 2023 Jul 28;12(8):1242. doi: 10.3390/antibiotics12081242 (PMC10451209; doi:10.3390/antibiotics12081242)
Supplement: Supplementary file 1 [file antibiotics-12-01242-s001.zip › antibiotics-2488300-supplementary.pdf]

antibiotics

MDPI

Table S1. Comorbidities

Disease	ICD-10 code
Respiratory tract infections	J00-J47
Asthma	J30, J45-46
Gastrointestinal disease	K20-K21, K25-K29, K58
Hypertension	I10-I15, O10-O16
Sexually transmitted infections	A51-52, A54-56, A59, A63, A74, O981, O982
Migraine or headache	G43-G44, R51
Urinary tract infections	N39, N34.1
Diabetes	E10-E14
Atopic dermatitis	L20
Renal disease	E112, E132, E142, I12-I13, N00-N08, N17-N19, N25-N27
Epilepsy	G40-G41
Drug dependence disease	F10-F16, F18-F19, Z71.4, Z71.5, Z72.1, Z72.2
Allergy	H101, T784, Z88,
